# Recurrence of Obstructive Sleep Apnea in Post-adenotonsillectomy Obese Pediatric Patients: A Systematic Review

**DOI:** 10.7759/cureus.84741

**Published:** 2025-05-24

**Authors:** Adi Cohen, Andrew Gunthner, Adam Cervone, Madison Uzwy, Stephanie N Urban-Galvez

**Affiliations:** 1 Medicine, Nova Southeastern University Dr. Kiran C. Patel College of Osteopathic Medicine, Davie, USA; 2 Pediatrics, Nova Southeastern University Dr. Kiran C. Patel College of Osteopathic Medicine, Davie, USA

**Keywords:** adenotonsillectomy, obese, obstructive sleep apnea, pediatrics, sleep disordered breathing, tonsillectomy

## Abstract

Obstructive sleep apnea (OSA) is an under-recognized consequence of pediatric obesity, with adenotonsillectomy (AT) serving as the standard first-line surgical treatment. In obese pediatric patients, OSA often recurs postoperatively, raising concern for the long-term efficacy of AT. This systematic review evaluates the recurrence of OSA in post-AT obese pediatric patients, with a focus on the key risk factors contributing to residual disease. A literature search was conducted using Embase, PubMed, and Web of Science. Eligibility criteria included peer-reviewed, English articles published within the last five years that contained pediatric patients less than 18 years old who had a body mass index (BMI) ≥ 95th percentile. All patients had to have been diagnosed with sleep-disordered breathing or OSA preoperatively and must have undergone AT. Eleven studies met these criteria, of which 82% demonstrated a significant association between obesity and recurrence of OSA following AT. Key factors included higher baseline apnea-hypopnea index, postoperative weight gain, and residual upper airway obstruction. Two studies contradicted this trend, but limitations such as the use of subjective questionnaires and short follow-up durations reduced the studies’ reliability. The findings indicate that AT alone is often insufficient in resolving OSA in obese pediatric patients. Given the high recurrence rates, a multidisciplinary approach, including weight management and non-invasive ventilation, may be needed to improve outcomes in this vulnerable population.

## Introduction and background

Obesity and its associated complications represent a significant portion of morbidity and mortality in the United States [1]. In particular, the rising incidence of childhood obesity in recent years has emerged as a critical public health crisis, imposing a substantial pediatric healthcare burden [2]. Currently, about 17% of children and adolescents are classified as obese with body mass index (BMI) ≥ 95th percentile, a condition that not only predisposes individuals to hypertension, coronary artery disease, and an elevated risk of developing type 2 diabetes and accompanying complications, but also leads to severe psychosocial repercussions [3,4].

An often-underappreciated consequence of obesity is obstructive sleep apnea (OSA), which affects nearly 60% of obese children [5]. OSA is defined as an intermittent obstruction of the upper airways during sleep, resulting in episodes of hypopnea or apnea, which impairs oxygenation and is associated with autonomic dysregulation and subsequent cardiovascular, pulmonary, and neurological sequelae [6]. Cardiovascular consequences specifically include systemic hypertension, coronary artery disease, and heart failure. Pulmonary and neurological manifestations are associated with pulmonary hypertension as well as memory and mood disturbances, respectively. Obesity is the most significant risk factor for OSA, in which children with obesity have three times higher odds of suffering from severe OSA than those who are not obese [7]. In pediatric populations, adenotonsillar hypertrophy represents an additional prominent risk factor [8,9]. Hypertrophy of adenoid and tonsillar tissues in the nasopharynx outpaces the growth of the surrounding bony structures, thereby reducing available airway space, leading to multiple nighttime awakenings which, in turn, contribute to higher rates of excessive daytime sleepiness, behavioral disturbances such as hyperactivity, and diminished cognitive outcomes [9,10].

In the United States, approximately 15% of pediatric surgeries involve tonsillectomy, the surgical removal of the tonsils, as an isolated procedure or in combination with the removal of the adenoids, classified as an adenotonsillectomy (AT) [11,12]. Historically, tonsillectomy was most commonly performed to treat conditions such as peritonsillar abscess, tonsilloliths, and chronic pharyngitis or tonsillitis. However, concerns regarding its efficacy, as well as the risks and potential sequelae associated with the procedure, have led to a significant decline in its use [13]. Moderate to severe pain, hemorrhage, respiratory compromise, nausea and vomiting, dehydration, and speech-related issues are plausible postoperative complications following tonsillectomy [14-16]. Animal studies have raised concerns regarding the potential detrimental effects of the procedure on the developing brain; however, it remains uncertain whether these findings are directly applicable to pediatric populations [17].

The rising prevalence of childhood obesity in recent years has become increasingly evident, presenting a new array of challenges for the medical community. One such challenge is OSA, a condition for which surgical intervention, particularly tonsillectomy, is often considered the primary treatment. However, like all surgical procedures, tonsillectomy carries inherent risks that may lead to postoperative complications. Furthermore, there are patients who continue to experience persistent or recurrent OSA following the procedure, calling into question the long-term efficacy of tonsillectomy. This paper aims to examine the recurrence of OSA in obese pediatric patients following AT.

## Review

Methods

Eligibility Criteria

Eligibility criteria were outlined to serve as a guideline for which articles were relevant to what our paper was aiming to prove. Articles that were published in English, peer reviewed, and published in the timeframe between January 2020 and January 2025 were included. Articles were limited to pediatric patients less than 18 years old who had a BMI ≥ 95th percentile for age and gender. One article included severe obesity, which is defined as BMI ≥ 120th percentile for age and gender. All patients had to have been diagnosed with sleep-disordered breathing or OSA preoperatively. These patients must have undergone AT by any method available. Outcomes were measured via polysomnography, drug-induced sleep endoscopy (DISE), and orthodontic/myofunctional evaluation after sleep clinical score. Studies compared pre- and post-procedural OSA variables to determine the recurrence of OSA.

Search Strategy

A systematic search was conducted to identify relevant studies using Embase, PubMed, and Web of Science. The following search string was inputted: (("Pediatric" OR "children" OR "adolescents" OR "infants") AND ("Sleep apnea" OR "obstructive sleep apnea" OR "OSA" OR "Sleep-disordered breathing" OR "SDB") AND ("Tonsillectomy" OR "Adenotonsillectomy" OR "Surgical removal of tonsils") AND (“obesity” OR “obese” OR “overweight”) AND ("Recurrence" OR "residual" OR "relapse" OR "post-operative outcomes" OR "polysomnography" OR "sleep study")).

Selection Process

Articles were downloaded as Research Information Systems (.ris) files from Embase and PubMed and Care Inspectorate Wales (.ciw) files from Web of Science. These files were uploaded to the online software Rayyan for screening and eligibility. A total of 426 articles were uploaded, 222 Embase, 82 PubMed, and 122 Web of Science. The article duplication detection tool embedded within the Rayyan platform was used to remove 152 duplicates, leaving 274 articles for title and abstract screening.

**Figure 1:**
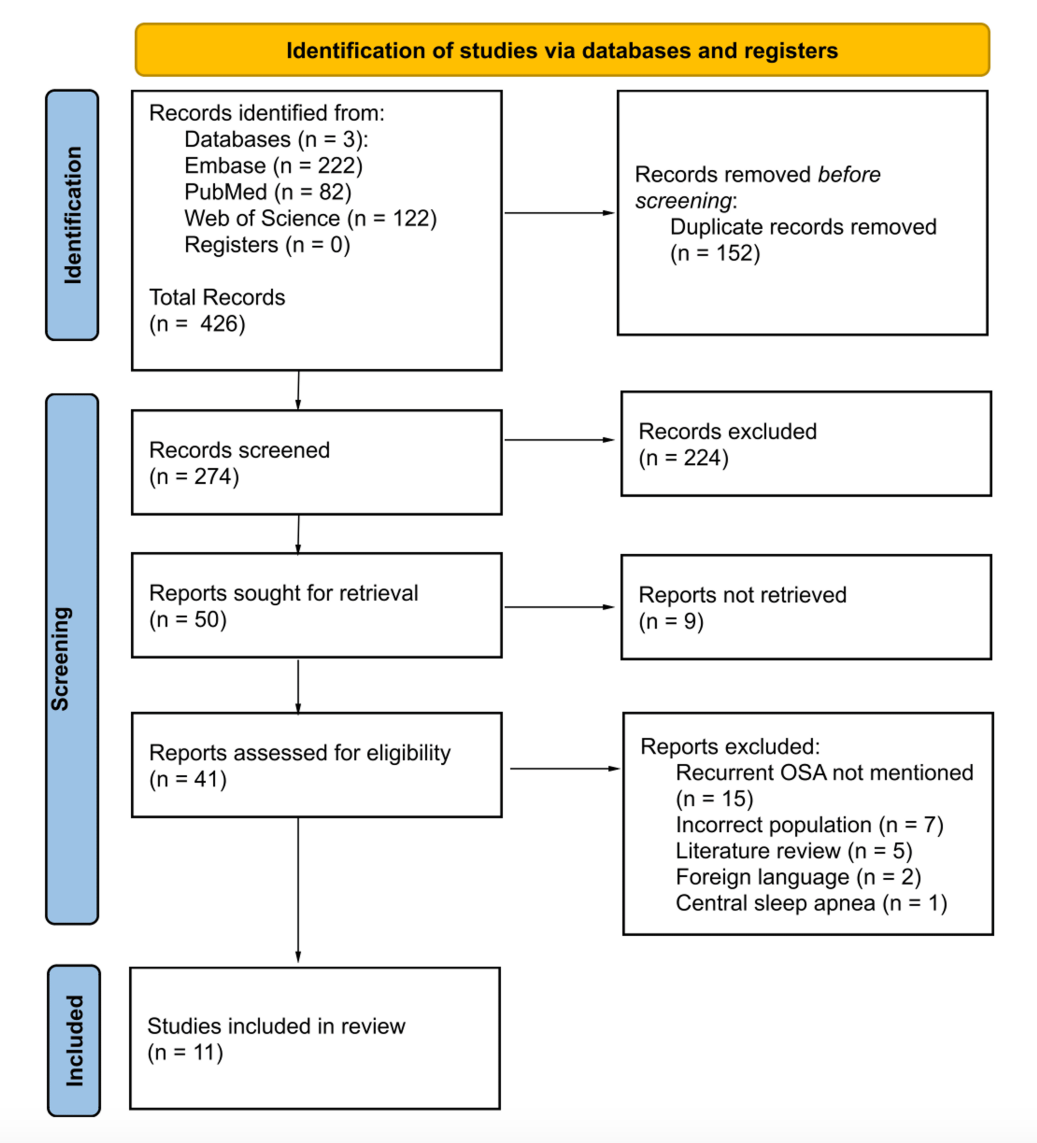
PRISMA flow diagram to summarize the screening process PRISMA: Preferred Reporting Items for Systematic Reviews and Meta-Analyses

The screening process began with title and abstract screening, in which two reviewers independently evaluated all 274 articles based on the eligibility criteria. A third reviewer served as the tie-breaker and resolved 73 conflicts after the conclusion of the title and abstract screening process. Two hundred and twenty-four articles were excluded for not satisfying the eligibility criteria; therefore, 50 articles progressed to full-text review. Initially, full texts were uploaded to the Rayyan software for review. Eleven articles were not retrieved for full text screening for reasons such as unavailable full-length text, discontinued journals, or non-English language. Two reviewers read through the full text of the available 39 articles to determine whether they met the eligibility criteria. A third reviewer served as the tie-breaker and resolved 24 conflicts after the conclusion of the full-text screening process. Twenty-eight articles were excluded while 11 articles were included in the systematic review. The screening process is summarized using the Preferred Reporting Items for Systematic Reviews and Meta-Analyses (PRISMA) flow diagram (Figure 1).

**Figure 2:**
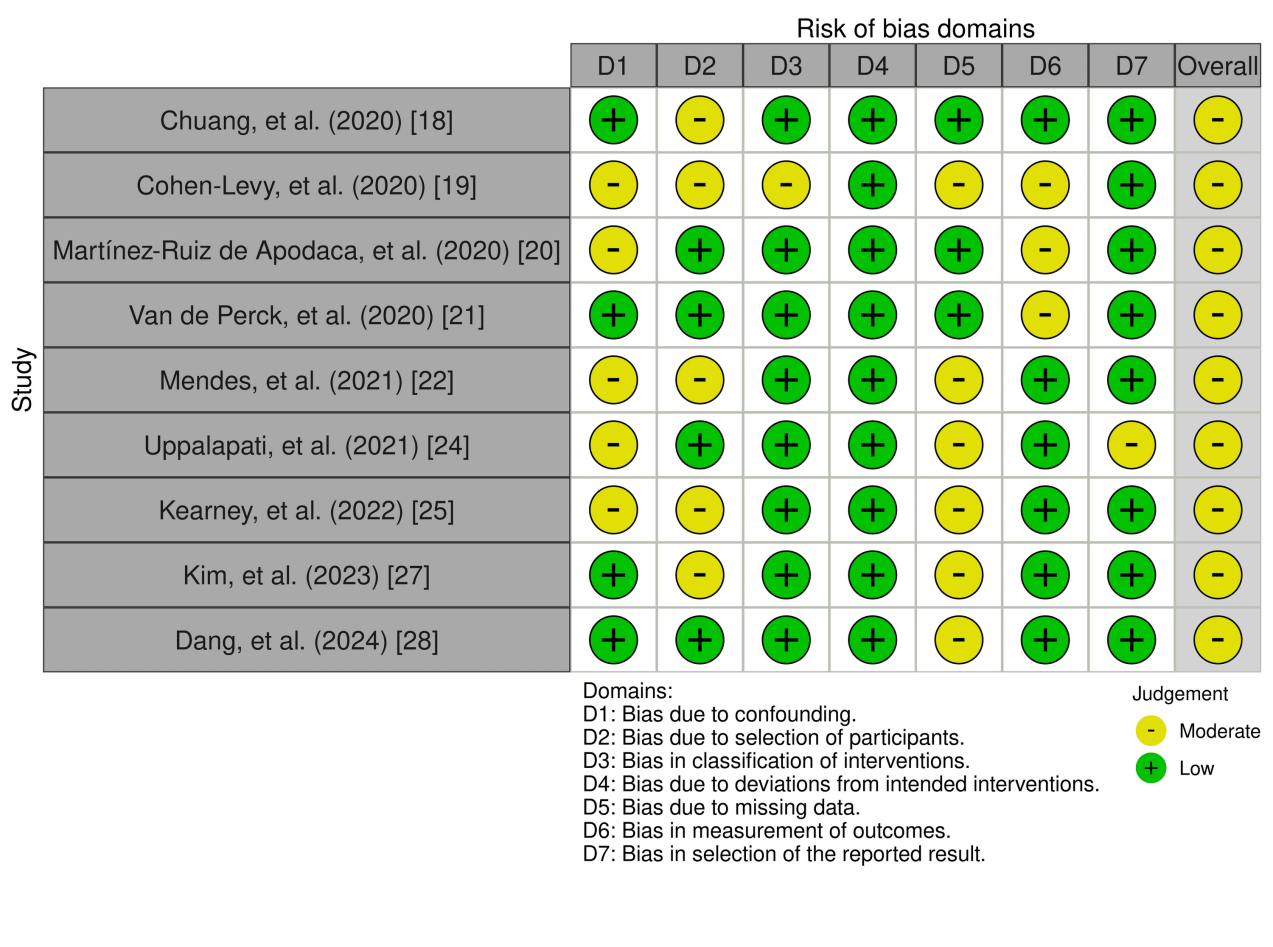
ROBINS-I tool to assess risk of bias ROBINS-I: Risk Of Bias In Non-randomized Studies - of Interventions

The ROBINS-I (Risk Of Bias In Non-randomized Studies - of Interventions) tool was used to assess the risk of bias within the studies analyzed (Figure 2) [18-28]. Two of the eleven included articles, including Fayson et al. [26] and Vakharia et al. [23], did not categorize as a non-randomized study and, as a result, could not be included in the ROBINS-I risk of bias tool.

Results

A total of 11 articles met the eligibility criteria focusing on the recurrence of OSA in post-AT obese pediatric patients. The criteria for diagnosing OSA across studies included polysomnography or clinical assessment with an Apnea-Hypopnea Index (AHI) greater than five events per hour. Obesity was additionally defined as BMI ≥ 95th percentile within the pediatric population being analyzed. The studies were organized by factors such as study design, sample size, and principal conclusions (Table 1). Of the 11 studies, six specifically investigated the effect of obesity on OSA recurrence following AT in children. The remaining five studies primarily focused on additional comorbidities, such as patient demographics, socioeconomic status, and craniofacial/orthodontic abnormalities with subsequent data on obesity that related to our topic of interest. The duration of follow-up post-AT ranged from three months to two years.

**Table 1:** Summary of included articles OSA: Obstructive Sleep Apnea; AT: Adenotonsillectomy; PSG: Polysomnography; ADI: Area Deprivation Index; AHI: Apnea-Hypopnea Index; SPSS: Statistical Package for the Social Sciences; DISE: Drug-Induced Sleep Endoscopy; SACS: Sleep Apnea Clinical Score; REI: Respiratory Event Index

Authors	Purpose	Study Design	Study Population	Methods	Limitations	Key Findings
Chuang et al. (2020) [18]	To explore the relationships between age, sex, weight status, disease severity, and inflammation levels in pediatric OSA patients and to develop a predictive model for surgical cure rates using established factors and inflammatory biomarkers	Prospective cohort study	60 Taiwanese children from Han ancestry with OSA (mean age - 7.5 +/- 2.2 years)	This study recruited children aged 5–12 with severe OSA for AT between March 2017 and January 2019, with informed consent obtained from parents and participants over six years old. PSG was conducted before and three months after surgery, and inflammatory biomarkers were measured to assess associations with OSA severity and surgical outcomes.	Selection bias due to the predominance of boys being studied, the lack of a normal control group, and only short-term outcomes were studied.	The study showed that there was an interdependent relationship between obesity and OSA.
Cohen-Levy et al. (2020) [19]	To investigate the prevalence of craniofacial/orthodontic abnormalities and oral dysfunctions in patients who had AT and continue to have OSA	Prospective observational study	100 patients with sleep-disordered breathing	4000 participants were invited, and only 100 were selected to represent the cohort for sleep-disordered breathing. Patients were invited to an orthodontic/myofunctional evaluation after the SACS, followed by a one-night sleep study.	There were a number of selected individuals who either declined or did not answer to being a part of the study.	Obese patients did not have more severe REI than lean patients.
Martínez-Ruiz de Apodaca et al. (2020) [20]	To investigate the efficacy of surgery on pediatric patients for OSA	Prospective cohort study	317 children (1-13 years old) with an AHI ≥3 with no previous treatment	Divided between surgical, medical, and observation, each patient underwent a PSG, exploration of the upper airway, and a questionnaire. Then 12 +/- 3 months a new PSG and questionnaire were given to each patient.	None	Being overweight or underweight was not related to therapeutic failure.
Van de Perck et al. (2021) [21]	To investigate the relationship between bodyweight to upper airway collapse and treatment outcomes	Retrospective cohort study	139 healthy children (>2 years old) diagnosed with OSA within a year	Retrospective analysis of previously collected PSG, DISE, and treatment outcomes of healthy children with OSA.	Retrospective design (no subject matching) and contained a small sample size	Circumferential collapse was more notable in obese children than in lean children. Cure rates for obese children after AT were 50% compared to lean children, which was 80.6%.
Mendes et al. (2021) [22]	To examine preoperative factors and their association with surgical failure or persistent OSA (AHI >5 after AT) in children with severe OSA, and assess the degree of airway collapse observed during DISE in instances of surgical failure and effectiveness of targeted surgery based on DISE results	Retrospective cohort study	80 patients with severe pediatric OSA	This retrospective study, conducted from August to September 2020, reviewed children with severe OSA treated at a hospital from 2011 to 2020. All underwent AT and repeat PSG three months post-surgery. Surgical failures had DISE for further planning. A chi-square test assessed the relationship between persistent OSA and preoperative characteristics.	Small sample size	Obesity was the strongest factor in surgical failure in children with severe OSA who had an AT.
Vakharia et al. (2021) [23]	To investigate the efficacy of AT in pediatric patients with severe obesity	Retrospective case series	9 pediatric patients (14 months to 13 years 4 months) with extreme obesity who underwent AT for OSA	A retrospective review of the clinic's patient database was conducted to identify individuals who underwent AT for the treatment of OSA.	Small sample size	AT in extremely obese pediatric patients has a far lower cure rate than the nonobese population.
Uppalapati et al. (2021) [24]	To investigate patients with severe OSA to see if there are differences in the outcomes in the surgery based on the initial severity of the OSA, comorbidities, or demographics	Retrospective cohort study	112 children (2-18 years old) with an AHI >10 who underwent AT and had a pre- and post-PSG	Bivariate analysis was conducted using the Pearson chi-square test. Univariate and multivariate analyses via binary logistic and multinomial linear regressions were performed using SPSS software.	Small sample size	It was found that there was a smaller reduction of AHI in obese patients and less of a lower cure rate of severe OSA in obese patients.
Kearney et al. (2022) [25]	To investigate the outcomes of AT in adolescents with obesity and OSA	Prospective cohort study	70 patients (12 to 18 years old) who underwent AT for OSA with pre- and postoperative PSG and 32 adolescents who were not obese and underwent AT for OSA with pre- and postoperative PSG	Adolescents with obesity and OSA who had AT done were compared to healthy nonobese adolescents who underwent AT for OSA and pre-/postoperative PSG.	Retrospective design (no subject matching), limited sample size, the sleep lab was unable to give data on oxygen desaturation index, and no follow-up data on patients' weight.	Patients with obesity were 7 times more likely to have moderate/severe persistent disease. Obese patients still had severe OSA compared to patients without obesity. After the AT, patients with obesity had higher AHI and arousal index compared to non-obese patients.
Fayson et al. (2023) [26]	To investigate whether there is an increase in OSA in Black children compared to non-Black children	Secondary analysis of a randomized controlled study	224 children (ages 5-9) with mild to moderate OSA who underwent AT	The outcome was residual OSA 6 months after the operation.	Only mild to moderate OSA was included, excluding severe OSA. Also included patients with parents who had time to undergo the study, therefore possible exclusion of "poorest and most socioeconomically challenged families"	Compared to nonobese children with OSA, obese children with OSA have worse AT response. More than half with residual OSA after the surgery were obese.
Kim et al. (2023) [27]	To investigate the connection between neighborhood level advantages and severe OSA in children	Retrospective case-control study	249 children (under the age of 18) who underwent AT followed by PSG within six months	The patients were placed into two groups based on the ADI.	A wide age range therefore the weight gain in puberty could have affected the weight parameters	AHI itself was not associated with severe or residual OSA. Yet, a lower socioeconomic neighborhood was associated with comorbidities that are linked to OSA. There was an association between severe OSA and obesity.
Dang et al. (2024) [28]	To investigate the relationship between weight gain after AT and the persistence of OSA	Retrospective cohort study	250 children (ages 2-17) who had AT with PSG and weight data before and after the surgery	The patient's perioperative weight and AHI index after the surgery were assessed and compared.	The lack of postoperative PSG studies available	There was an increase in residual OSA with an increase in perioperative weight.

It can be concluded that there is a definitive association between obesity in pediatric patients and the recurrence of OSA post-AT in a majority of the included studies. The AHI was a principal measurement that quantified the relationship between the variable of interest (obese) and control (non-obese). In studies where recurrence was documented, it was found that children with a higher BMI had persistently elevated AHI post-AT and, therefore, a greater likelihood of recurrence of OSA. One such study found that "post-AT, children with obesity had a higher AHI (P < 0.001) and arousal index (P = 0.011) when compared to the nonobese group” concluding that there is a seven times greater likelihood of moderate to severe persistent OSA (odds ratio = 7.1, 95% confidence interval [2.24, 22.48], P = 0.001) following surgery for pediatric patients with obesity than those without obesity [25]. Another study performed a univariate linear regression analysis of percent reduction in AHI after AT and concluded that obesity was associated with a statistically significant smaller percentage reduction in AHI after AT with an obese 95% CI coefficient at -14.535 and p-value of 0.001 [24].

Another predictor of recurrence of OSA post-AT was the presence of epiglottis collapse and adenoid tissue in obese children, which was evaluated in two of the included studies. One study investigated these variables as most notable in obese pediatric patients leading to “cure rates of 80.6% in lean children, 100% in overweight children, and 50.0% in obese children (Fisher's exact: P ¼ 0.064)" [21]. Another study mentioned these variables as the most prevalent in postoperative drug-induced endoscopy of children with persistent OSA, concluding that the most notable predictor in AT surgical failure in children with severe OSA is obesity [22].

Some studies within the review compared post-AT residual OSA with differences in BMI but fell below the obesity threshold. Nonetheless, it was found that residual OSA was more persistent in pediatric patients with an average BMI of 27.7 compared to a BMI of 20.9 (Pearson x2 test = 0.001) [28]. Another study examining similar metrics found that residual OSA presented in patients with an average BMI of 27.2 which is higher than those without residual OSA at a BMI of 24.4 (Pearson x2 test = 0.146) [27]. Although these studies contain limitations in terms of their obesity metric and statistical significance, the articles found a notable difference when comparing obese and non-obese pediatric patients with residual OSA, warranting further evaluation.

Two studies that abided by the eligibility criteria of the systematic review disproved our research topic in question. One such study stated that "BMI, BMI-percentile, or being in the obesity (>95% percentile) or underweight ranges (<5% percentile), were not related to therapeutic failure" [20]. These variables were dependent on the responses to the Pediatric Sleep Questionnaire, one of the most commonly used questionnaires for clinical screening of childhood sleep breathing disorders [29]. However, the questionnaire was administered three months post-AT and relied on the patient and patient’s family reporting of ongoing symptoms, posing a considerable bias toward the result. The other study stated that “obese participants did not have more severe Respiratory Event Indexes (REIs) than lean patients in this sample when comparing both BMI z-scores (1.82 ± 2.65 for < 2 standard deviations vs 1.70 ± 1.42 for ≥ 2 standard deviations) [19]. A notable weakness of this study was the retrospective collection of surgical data, which could not provide precise assessment of obesity status prior to surgery [19].

In all, nine out of the eleven (82%) studies that were investigated in this systematic review supported our hypothesis that there is a recurrence of sleep apnea in post-AT obese pediatric patients. Even the studies that focused on other comorbidities as their principal variable of interest found a statistically significant connection between obesity and OSA stating that of those that had residual OSA within their cohort, 64% were obese with a p-value of 0.001 [26]. Another study found that at three months post-AT, patients with obesity with non-severe OSA had 36% surgical cure rate and patients with obesity with severe OSA had 46% cure rate compared to nonobese non-severe OSA that had a 75% surgical cure rate and nonobese severe OSA had 82% cure rate with a statistically significant p-value of 0.03 [18]. The risks and benefits should, therefore, be weighed when evaluating whether such an invasive surgical procedure should be used to cure OSA in this population.

Discussion

Interpretation of Results

The primary aim of this study was to investigate the degree of OSA recurrence in obese pediatric patients post-AT. Our findings provide strong evidence that a significant proportion of these children experience recurrence of OSA. The articles suggest that while AT may be an efficacious treatment for non-obese children with OSA, its effectiveness in obese children is limited, with many studies describing poorer surgical outcomes and higher likelihood of disease recurrence. This suggests that it may be more beneficial for obese patients to reduce their BMI prior to being considered a surgical candidate. In patients who strictly suffer from obesity, where weight-induced airway compression is more prominent, there may be little evidence to support that AT is helpful.

A key finding in this review was the association between obesity and the recurrence of OSA after AT in pediatric patients. Obese children were found to have a significantly higher likelihood of having moderate to severe persistent OSA after AT, as evidenced by Kearney et al., demonstrating a seven-fold increase in the odds of OSA recurrence in adolescents with obesity than their non-obese counterparts [25]. The Arousal Index also indicated that children with obesity had a greater frequency of arousals during sleep post-surgery [25]. This further supports the idea that the recurrence of OSA in these children is not a minor postoperative complication, but a much greater ongoing health concern.

Another important factor linked to the recurrence of OSA in this review is the presence of anatomical abnormalities such as epiglottis collapse and residual adenoid tissue. Other studies, such as that by Van de Perck et al., suggest that the tonsils and adenoids may not be the sole determinants of OSA in obese children. Van de Perck et al. found that there were greater rates of upper airway circumferential collapse in obese children than in normal weight children [21]. This anatomical variation highlights the importance of conducting a thorough preoperative evaluation in pediatric cases of OSA with concurrent obesity. Although AT addresses the tonsils and the adenoids, these additional findings in obese children may require a more tailored surgical approach.

Clinical Implications

When analyzing the results of this review, the findings suggest that AT may not be as effective in treating OSA in obese pediatric patients compared to their non-obese counterparts. For instance, it was found that those who had residual OSA required ongoing use of non-invasive ventilation [23]. The persistence of OSA may require physicians to take another path of care focused on individualized treatment plans. Physicians may need to reassess whether it is beneficial for obese pediatric patients to be candidates for AT and rather transition to an emphasis on non-surgical interventions or ones that combine both medical and holistic approaches. This can include a focus on weight management including nutritional modifications and exercise, sleep hygiene, and allergy management; nonetheless, more research is needed to determine the most beneficial modality of care. Given that the studies showed a persistence in OSA in obese pediatric patients post-AT, there needs to be close monitoring and long-term follow-up in this particular group through scheduled laboratory sleep studies or home sleep apnea tests.

Limitations in the Review Process

During the process of this review, there were some limitations in the collection and interpretation of reliable data. This review was limited to three databases due to accessibility, yet broadening the search to include additional databases might have yielded more articles for analysis. The studies in this review were diverse regarding the design, sample size, and follow-up duration, which complicates the ability to draw conclusive evidence about the relationship between childhood obesity and OSA recurrence post-AT. Differences in diagnostic methods (polysomnography, questionnaire, clinical assessments) across studies may have contributed to variability in the results. Furthermore, two of the eleven articles, including Fayson et al. and Vakharia et al., were not categorized as a non-randomized study and, as a result, could not be included in the ROBINS-I risk of bias tool. In the article by Fayson et al., the RoB (Risk of Bias) 2 tool was used which concluded low risk of bias; however, the article by Vakharia et al. is a retrospective case series in which a risk of bias cannot be performed [23,26].

Future Research

Future research should be focused on conducting large-scale studies with extended follow-up periods focused on investigating the specific mechanism by which obesity contributes to the recurrence of OSA postoperatively, such as the effects of excess adipose tissue on airway anatomy. Additionally, while the review suggests that obesity negatively affects AT outcomes, it remains unclear whether weight loss prior to or following surgery can reduce OSA persistence. With the increased use of GLP-1 analogs as a means of weight loss, a future area of research could compare whether pharmacological treatment or lifestyle modifications can enhance the effectiveness of AT. Though many obese patients suffer from adenotonsillar hypertrophy, more research clarifying outcomes of AT in obese patients with and without AT hypertrophy would present an entirely new question.

## Conclusions

The key findings of the literature analysis suggest a notable recurrence of OSA in post-AT obese pediatric patients. Despite the removal of tonsils in children, the superimposed risk factor of obesity serves too substantial a role in the rate of complications and disease recurrence. The evidence indicates that while AT may provide minimal reduction in sleep apnea parameters, such as the AHI and REI, there are limitations to the complete resolution of OSA in this vulnerable population. These findings highlight the need to reevaluate the treatment protocol for managing OSA in obese children. The risks vs. benefits of surgical intervention are affected by a lack of resolution of OSA, which should be taken into consideration. Additional therapeutic interventions and long-term management should address the underlying risk factors contributing to recurrence of OSA to provide multidisciplinary care for obese pediatric patients with OSA.
